# One-step synthesis of pyridines and dihydropyridines in a continuous flow microwave reactor

**DOI:** 10.3762/bjoc.9.232

**Published:** 2013-09-30

**Authors:** Mark C Bagley, Vincenzo Fusillo, Robert L Jenkins, M Caterina Lubinu, Christopher Mason

**Affiliations:** 1 Department of Chemistry, School of Life Sciences, University of Sussex, Falmer, Brighton, East Sussex, BN1 9QJ, UK; 2 School of Chemistry, Main Building, Cardiff University, Park Place, Cardiff, CF10 3AT, UK; 3 CEM Microwave Technology Ltd, 2 Middle Slade, Buckingham, MK18 1WA, UK

**Keywords:** Bohlmann–Rahtz, continuous flow processing, ethynyl ketones, flow chemistry, Hantzsch dihydropyridine synthesis, heterocycles, microwave synthesis, multicomponent reactions, pyridine synthesis

## Abstract

The Bohlmann–Rahtz pyridine synthesis and the Hantzsch dihydropyridine synthesis can be carried out in a microwave flow reactor or using a conductive heating flow platform for the continuous processing of material. In the Bohlmann–Rahtz reaction, the use of a Brønsted acid catalyst allows Michael addition and cyclodehydration to be carried out in a single step without isolation of intermediates to give the corresponding trisubstituted pyridine as a single regioisomer in good yield. Furthermore, 3-substituted propargyl aldehydes undergo Hantzsch dihydropyridine synthesis in preference to Bohlmann–Rahtz reaction in a very high yielding process that is readily transferred to continuous flow processing.

## Introduction

Microwave-assisted synthesis has revolutionized many processes in recent years as a valuable alternative to the use of conductive heating for accelerating transformations in synthetic organic chemistry [1], colloidal science [2], natural product chemistry [3], medicinal chemistry [4–6], solid-phase peptide synthesis [7] and in the biosciences [8]. Despite the many advantages of this heating method, and the introduction of a wide range of instrumentation [1], the scale up of microwave-mediated reactions still poses a number of challenges, in particular as a result of a lack of uniform heating [9]. Scale-up using batch methodologies in open reaction vessels can give excellent yields but might not be appropriate for certain volatile or toxic reagents whereas continuous flow processing, providing the reaction mixture is homogeneous, allows transfer from small-scale sealed vessel conditions to mesoscale production often without any modification of reaction conditions or loss in product yield [10]. The transfer from microwave batch reaction to continuous flow processing can offer many advantages for scale up, certainly in terms of process intensification or in combination with reagent and scavenger cartridges for multi-step synthesis [11], and is possible using conventionally heated micro- or mesofluidic flow devices [12–13], but is also feasible under microwave dielectric heating. Strauss first demonstrated in 1994 that by combining microwave-heating technology with continuous flow processing, problems with the limited penetration depth of microwave irradiation and the physical restrictions of a standing wave cavity could be overcome [14]. A continuous flow reactor has the potential for rapid optimization using minimal quantities of reagents, and for ‘scaling out’ – the spatial resolution of reactants and products can, in principle, sustain indefinite production [15]. Following Strauss’s original report, a variety of transformations have been described using this unique combination of microwave heating and continuous flow or stop-flow processing [16–17]. Continuous flow microwave reactors have been used in transition metal-mediated cross-coupling reactions by Organ et al. in a Pd-coated capillary [18–20], over a solid-supported Pd catalyst using a thin layer of gold as a selective heating element [21], or in a Pd-supported silica monolith flow reactor by Haswell et al. [22], using palladated Raschig rings in a PEEK [poly(ether ether ketone)] reactor by Kirschning et al. [23], in a de novo glass coiled flow cell by researchers at Boehringer Ingelheim [24] and with an encapsulated palladium catalyst by Baxendale and Ley, et al. [25], the latter to process multigram quantities in a microwave-assisted Suzuki–Miyaura coupling. A comparison on the use of palladium(0) nanoparticle catalysts on glass-polymer composite materials in batch and flow-through experiments by Kappe, Kunz and Kirschning revealed that continuous flow processing gave better conversions and improved catalyst recycling, with no loss of activity [26]. A range of other applications have been explored, from a continuous flow isothermal narrow channel microreactor for process intensification of benzyl alcohol oxidation [27], the esterification of benzoic acid in a microwave tubular flow reactor [28], a continuous flow recycle microwave reactor for homogeneous and heterogeneous processes [29], a mesoscale flow reactor utilizing Fe_3_O_4_ as a microwave absorbing packed reactor bed with internal fibre optic temperature measurement [30], to the continuous flow preparation of biodiesel on large scale [31], processing up to 7.2 L min^–1^, and waxy ester production on pilot scale using a continuous microwave dry-media reactor [32]. The introduction of proprietary instruments capable of carrying out microwave-assisted transformations under flow processing have greatly expanded the range of chemistries scaled up and evaluated using this technology [1,9,16,33–42]. With all of these developments, it is becoming increasingly clear that flow chemistry, and to some degree microwave flow chemistry, is realizing its potential towards the next evolutionary step in synthetic chemistry [43].

**Scheme 1:**
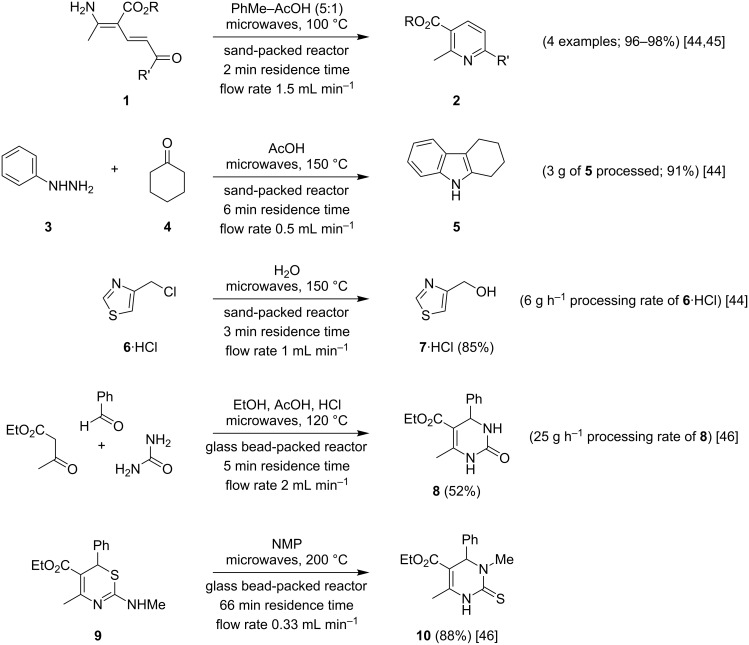
Mesoscale production of heterocycles in a continuous flow microwave reactor [44–46].

In 2005 we described a new continuous flow reactor design for microwave-assisted synthesis that operates in the optimum standing-wave cavity of a proprietary instrument [44]. The principal features exhibited by this reactor, charged with sand to produce a series of microchannels, included improved performance over a Teflon coil reactor, heated using the same single-mode instrument, and direct measurement of the flow cell temperature using the instrument’s in-built IR sensor. In a range of synthetic transformations (Scheme 1), including Bohlmann–Rahtz cyclodehydration of aminodienones **1** to the corresponding pyridines **2** [44–45], Fischer indole synthesis of tetrahydrocarbazole **5** from phenylhydrazine (**3**) and cyclohexanone (**4**) [44], and hydrolysis of 4-chloromethylthiazole (**6**) to give the corresponding alcohol **7** [44], the transfer from batch reactor operation to continuous flow processing was efficient and required little further optimization. Furthermore, we showed that methodology developed using different reaction platforms, including commercial microreactors and stainless steel continuous flow instruments, transfer well to our de novo microwave flow cell and from there can be scaled up using a commercial microwave flow reactor for mesoscale production [45]. The basic design of our microwave flow cell has been adapted by Kappe for the synthesis of dihydropyrimidinone **8** in a 3-component Biginelli reaction and for the preparation of N3-substituted dihydropyrimidinone **10** by Dimroth rearrangement of 1,3-thiazine **9** [46]. In these studies, the 10 mL flow cell was loaded with glass beads and irradiated at 120 or 200 °C, respectively, to give the target heterocycle in yields that compared very favourably with microwave-heated batch experiments. For dihydropyrimidinone **8**, a flow rate of 2 mL min^–1^ delivered a very respectable processing rate of 25 g h^–1^.

Following the success of this reactor design in delivering pyridine and pyrimidine heterocycles, albeit from very different processes, and the recent advent of new technology for mesoscale microwave-assisted continuous flow reactions [30], we set out to establish if readily-available ethynyl carbonyl precursors were capable of delivering diverse heterocyclic targets under a continuous flow regime under microwave heating. Ley and Baxendale et al. [47] have demonstrated that ethynyl ketones can be generated in flow by the palladium-catalysed acylation of terminal alkynes and further transformed in a continuous process to pyrazoles by cyclocondensation with hydrazines using a commercially available conductive heating modular flow reactor. Given that this cyclocondensation proceeds in a similar fashion and high efficiency under microwave irradiation [48], and that we have previously demonstrated that pyridines and pyrimidines can both be formed rapidly and efficiently from ethynyl ketones using microwave dielectric heating, the transfer of synthetic procedures to a continuous flow processing regime in a microwave flow reactor seemed highly feasible to access pyridine derivatives in a single step.

## Results and Discussion

### Synthesis of pyridines in a continuous flow reactor

Many of our previous studies on the synthesis of pyridines in a continuous flow reactor examined the cyclodehydration of Bohlmann–Rahtz aminodienone intermediates in the presence of a Brønsted acid catalyst [44–45]. This relatively simple cyclization reaction was utilized previously as we had already established its facility under microwave irradiation and so it provided a good comparison of different technology platforms. If the cyclodehydration could be incorporated into a multi-step process and was spontaneous under the reaction conditions, following Michael addition to ethynyl ketones, then the continuous production of pyridines from readily-available materials could be realized.

#### Introduction to the Bohlmann–Rahtz pyridine synthesis

**Scheme 2:**
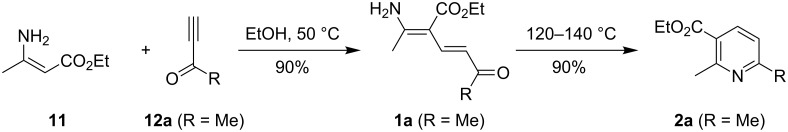
The original Bohlmann–Rahtz synthesis of pyridines [49].

Bohlmann and Rahtz first reported the synthesis of trisubstituted pyridines from a stabilized enamine, such as ethyl β-aminocrotonate (**11**), and an ethynyl carbonyl compound, such as butynone (**12a**), in 1957 [49]. In its original form it was a two step procedure involving Michael addition, isolation of the corresponding aminodiene intermediate (e.g. **1a**) and subsequent cyclodehydration under high temperature conditions neat under vacuum to give a 2,3,6-trisubstituted pyridine (**2a**: Scheme 2) with total regiocontrol. In recent years there has been renewed interest in this transformation for its application in target synthesis [50], in the development of one-pot procedures for pyridine synthesis [50–58], and for incorporation into domino processes [57–59]. Given our precedent that microwave irradiation can facilitate the one-pot Bohlmann–Rahtz synthesis of pyridines from ethynyl ketones [58–60], this reaction was an ideal starting point to investigate the synthesis of pyridines under a continuous flow regime, from which a comparison to other methods could be drawn.

#### Bohlmann–Rahtz pyridine synthesis in batch and flow

**Scheme 3:**
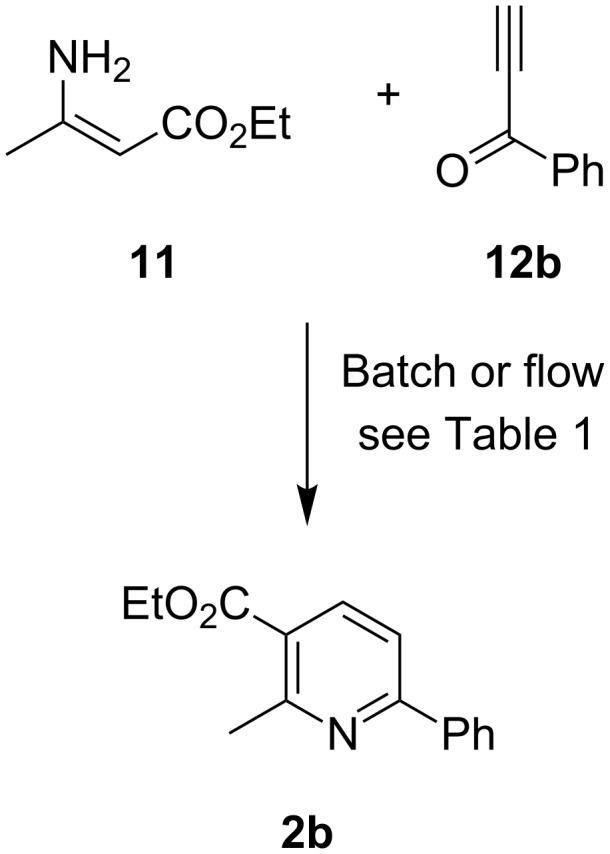
Bohlmann–Rahtz synthesis of pyridine **2b**.

The reaction conditions, temperature and residence time were optimized in batch mode under microwave irradiation for the one pot synthesis of pyridine **2b** (Scheme 3) using ethyl β-aminocrotonate (**11**) and a readily available ethynyl ketone, phenylpropynone **12b** (R = Ph) [51], in the presence of acetic acid as a Brønsted acid catalyst for transfer to flow processing. A range of conditions were investigated (Table 1) and, in each case, ^1^H NMR spectroscopic analysis of the crude reaction mixture revealed if unreacted starting materials were present. Microwave irradiation of a 1:1 ratio of starting materials **11** and **12b** at 100 °C for 15 min (hold time) in PhMe–AcOH (5:1 *v*/*v*) and spectroscopic analysis of the crude reaction mixture showed the two-step-in-one synthesis of pyridine **2b** was a success (Table 1, entry 1). Cyclodehydration was spontaneous under the reaction conditions as no aminodienone intermediate **1b** (R = Ph) was observed, although some unreacted propynone remained (**2b**:**12b** ~13:1). The use of a small excess of enamine **11** (1.3 equiv) and extending the reaction time to 20 min, improved the product ratio (Table 1, entry 2). At a higher reaction temperature, consumption of reactants was complete in 5 min on irradiation at 140 °C in PhMe–AcOH (5:1) (Table 1, entry 3) or at 120 °C in EtOH–AcOH (5:1) (Table 1, entry 4), to give pyridine **2b** in 74 or 86% isolated yield, respectively. The use of ethanol as a protic solvent appeared to improve the efficiency of the process, a phenomenon that has also been observed for two-step-in-one Bohlmann–Rahtz reactions [54], in the Michael addition of ethynyl ketones [51], both under conductive heating, and in the tandem oxidation Bohlmann–Rahtz synthesis of nicotinonitriles under microwave irradiation [59]. These batch experiments now established that microwave heating could establish efficient conversion to nicotinoate **2b** in 5 min using only a small excess of enamine **11** and so these parameters (Table 1, entry 4) were favoured for transfer to flow processing over previously reported procedures [58].

**Table 1:** Batch and flow experiments for Bohlmann–Rahtz synthesis of pyridine **2b**.

Entry	Process	Heating	Conditions^a^	Results^b^

1	Batch	Microwaves^c^	PhMe–AcOH, 100 °C, 15 min	**2b**:**12b** ~13:1^d^
2	Batch	Microwaves^c^	PhMe–AcOH, 100 °C, 20 min	**2b**:**12b** >95:5^d^
3	Batch	Microwaves^c^	PhMe–AcOH, 140 °C, 5 min	**2b** (74%)
4	Batch	Microwaves^c^	EtOH–AcOH, 120 °C, 5 min	**2b** (86%)
5	Flow	Microwaves^c^	EtOH–AcOH, 120 °C, 5 min	**2b** (76%)
6	Flow	Conductive^e^	EtOH–AcOH, 120 °C, 5 min	**2b** (86%)
7	Flow	Conductive^e^	EtOH–AcOH, 120 °C, 5 min	**2b** (71%)
8	Batch	Microwaves^f^	EtOH–AcOH, 120 °C, 5 min	**2b**^d^ (32%)
9	Batch	Microwaves^f^	EtOH–AcOH, 100 °C, 2.5 min	**2b**^d^ (28%)

^a^Reagents (0.3 mmol) were used in a molar ratio (**11**:**12b**) of 1:1 (entry 1) or 1.2:1 (entries 2–7) in PhMe–AcOH or EtOH–AcOH (5:1 *v*/*v*); temperature refers to vessel temperature, maintained by moderation of the initial microwave power (120 W for experiments in PhMe, 90 W for batch experiments in EtOH and 100 W using the flow cell), as measured by the in-built IR sensor (entries 1–5); ^b^outcome determined by ^1^H NMR spectroscopic analysis of the crude reaction mixture; numbers in parentheses refer to the isolated yield of pyridine **2b**; ^c^carried out using a commercial CEM single-mode instrument; ^d^unreacted starting materials were present; ^e^carried out using a commercial Uniqsis FlowSyn stainless steel coil reactor at a flow rate of 1 mL min^–1^ (5 mL reactor; entry 6) [60] or 4 mL min^–1^ (20 mL reactor; entry 7); ^f^the scaled up microwave-assisted reaction was carried out in a 60 mL Teflon vessel in batch mode using a commercial Milestone multi-mode instrument in a molar ratio (**1a**:**2b**) of 1.3:1 (15 mmol).

**Figure 1:**
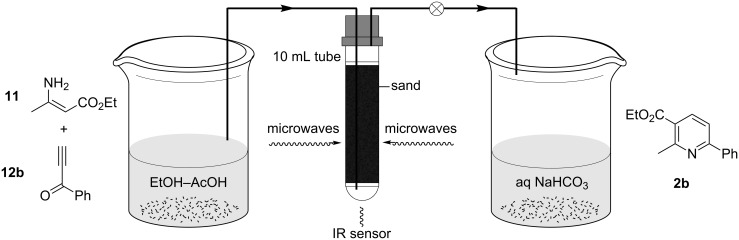
Microwave flow reactor for the Bohlmann–Rahtz synthesis of pyridine **2b**.

Following the success of the microwave batch reaction conditions, the most efficient parameters were transferred to the microwave flow reactor for continuous processing (Figure 1). The Pyrex tube was filled with sand, connected to a back-pressure regulator (100 psi) and primed with solvent at a flow rate of 0.6 mL min^−1^ (for 5 min residence time) using a HPLC pump. Microwave irradiation under continuous flow processing was initiated at an initial power of 100 W, which was modulated to maintain 120 °C vessel temperature as measured by the in-built IR sensor. Once the flow cell temperature stabilized, the solution of the reactants was introduced and the cell was irradiated at 120 °C for 5 min. Once all of the reactants were processed, the flow cell was washed with further batches of solvent and the outflow was quenched in a solution of aqueous NaHCO_3_. After extraction and purification by column chromatography, pyridine **2b** was isolated as a single regioisomer in 76% yield (Table 1, entry 5) and comparable purity to the successful batch experiments. By carrying out both Michael addition and cyclodehydration in one continuous flow process, pyridine synthesis is possible in a single step from readily available materials, avoiding the need to isolate and purify Bohlmann-Rahtz intermediate **1b** and overcoming issues of its poor solubility, which in past reports have necessitated carrying out the flow process under high dilution conditions [44–45].

With successful transfer of parameters to the microwave flow reactor, flow experiments were investigated with a commercial conductive heating flow reactor (Table 1) using either a 5 mL [60] (Table 1, entry 6) or 20 mL (Table 1, entry 7) stainless steel coil reactor. Both processes gave efficient conversion to pyridine **2b** with small variations noted depending upon the size of the flow cell – the smaller reactor (5 mL) and slower flow rates delivering the highest efficiency (Table 1, entry 6). The isolated yield of the continuous flow process (86%) outperformed the traditional Bohlmann–Rahtz reaction [49] in terms of overall yield (81% over two steps) and step efficiency. Furthermore, the process was comparable in efficiency to previously reported two-step-in-one Bohlmann–Rahtz methods under conductive heating [51], such as heating at 50 °C for 6 h in PhMe–AcOH (85% yield) or heating at reflux in PhMe for 5.5 h in the presence of zinc(II) bromide (15 mol %) (86% yield), and provides improvements in reaction kinetics and processing rate.

Finally the improved performance of flow processing in this transformation was validated by a larger scale (15 mmol) microwave-assisted batch reaction in a 60 mL Teflon vessel using a commercial multi-mode instrument (Table 1, entries 8 and 9). Irradiation at 120 °C for 5 min (Table 1, entry 8), in this case as measured by thermocouple, gave multiple components by tlc analysis and provided pyridine **2b** in poor isolated yield (32%) although did allow rapid access to gram quantities of material. In case a lack of homogeneity in heating had caused additional decomposition, the experiment was repeated over a shorter reaction time at a slightly lower temperature (Table 1, entry 9) but this caused no improvement in outcome (28% yield). Difficulties in scaling up a microwave-assisted batch reaction had been noted previously for the Bohlmann–Rahtz cyclodehydration [45]; in that case the use of a 60 mL vessel (1.9 mmol of **1b**) in a multi-mode instrument had proved highly efficient (96%) under similar conditions. However, transfer to a 100 mL vessel (7.7 mmol of **1b**) had caused a considerable decrease in efficiency (65%). For the two-step-in-one Bohlmann–Rahtz reaction these difficulties seemed to be even more pronounced: given the poor yield of the two processes (Table 1, entries 8 and 9), these difficulties were attributed to low efficiency in the initial Michael addition. For this multistep process, reaction parameters did not transfer well between single-mode 10 mL and multi-mode 60 mL reactors and this justified the use of continuous flow processing for efficient mesoscale production. It is evident that the reliability of scaling a microwave batch reaction is highly dependent upon the nature of the transformation and even small changes in the process in question can cause unexpected problems, which require further optimization of parameters to resolve. From these considerations, we conclude that the most reliable means to scale to gram production from mg scale in a microwave-assisted batch reaction using 10 mL sealed vessels is through continuous flow processing, using either microwave dielectric heating or conductive heating, which in this case gave comparable results.

### Synthesis of dihydropyridines in a continuous flow reactor

#### Introduction to Hantzsch dihydropyridines

The Hantzsch dihydropyridine (DHP) synthesis, first discovered in 1881 [61–62], is a well-studied multicomponent reaction, that provides structures with well-catalogued clinical properties for the treatment of cardiovascular disease, thrombosis and atherogenesis [63–66]. The 4-component process has been carried out under high temperature conditions in an autoclave [67] and under microwave irradiation [10,68–72]. Furthermore, this reaction has been studied in a conductive heating Uniqsis FlowSyn reactor [71,73] and its comparison with microwave heating batch experiments showed that the energy efficiency of these technology platforms vary with scale, but broadly are comparable [73]. Furthermore, recently a bespoke microwave reactor with a glass containment cell has been used under continuous flow processing for 4-component Hantzsch DHP synthesis in good yield [74]. Given this precedent and our own previous studies on the use of microwave irradiation in a single-mode instrument to promote 4-component DHP synthesis [70], this reaction seemed ideal to expand the scope of the microwave flow cell. Mechanistically, there was evidence to support the hypothesis that the 4-component Hantzsch reaction [64] proceeds in a similar course to the Bohlmann–Rahtz pyridine synthesis [50], by Michael addition followed by cyclodehydration, and so it was reasonable to assume that similar conditions should enable the flow processing of material, provided that reactants and products were homogeneous in the solvent of choice, thus providing further comparative studies on the transfer of parameters between different platforms.

#### Hantzsch dihydropyridine synthesis in batch and flow

**Scheme 4:**
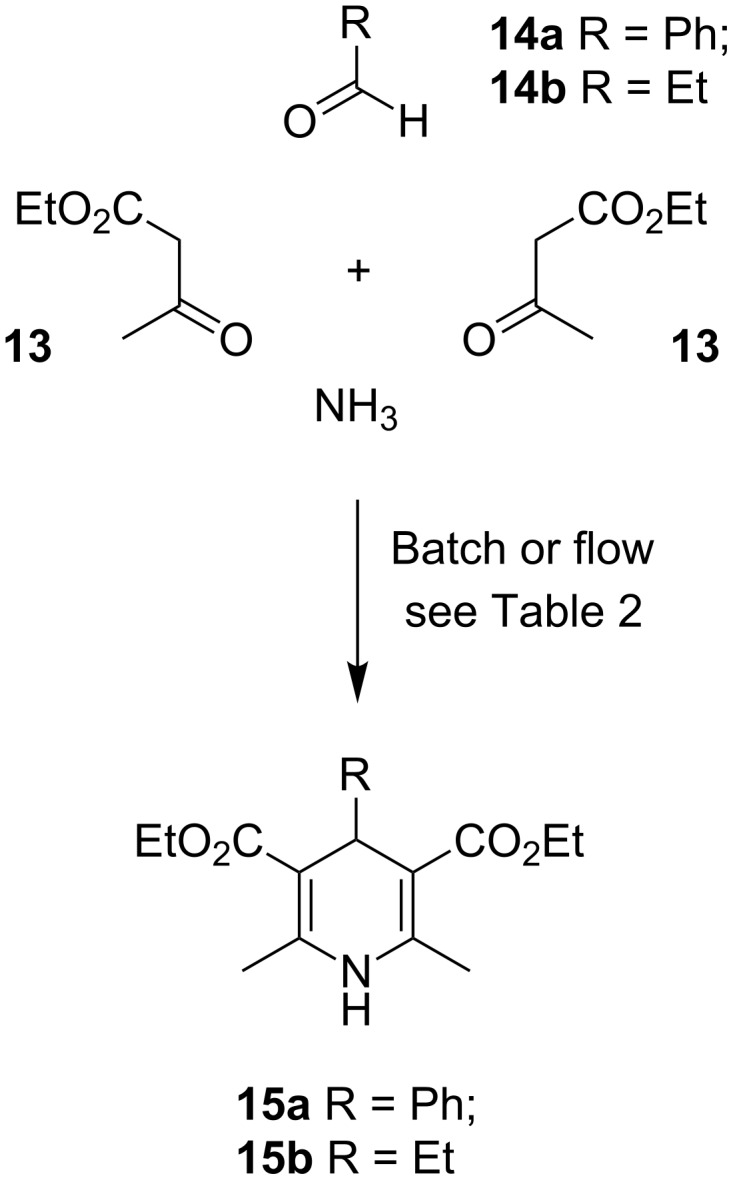
Four-component synthesis of Hantzsch DHP **15a,b**.

Previous methods for carrying out the microwave-assisted batch reaction were first consolidated by setting up a series of reactions that were purified in a consistent fashion. A solution containing an excess of ethyl acetoacetate (**13**), aqueous ammonia as the ammonia source, and either benzaldehyde (**14a**) or propionaldehyde (**14b**) (Scheme 4) was irradiated at 140 °C for 10 min in EtOH–H_2_O (1:1 *v*/*v*) in a modification of the Leadbeater conditions [73] (Table 2, entries 1 and 2). The outcome was compared with a repeat of our previously established conditions [70], based upon Westman’s report [68], in EtOH (Table 2, entries 3 and 4), in all cases purifying by flash chromatography on silica. For the synthesis of phenyl-**15a** (70% yield) and ethyl-DHP **15b** (87% yield) on 2.5 mmol scale these experiments indicated that the ideal solvent for this process was EtOH rather than EtOH–H_2_O (1:1 *v*/*v*). Reducing the molar equivalents of acetoacetate **13** from 5 to 3.4 (the stoichiometry used by Leadbeater [73]) caused a significant reduction in the yield for both reactions (Table 2, entries 5 and 6) and so did justify the use of such a considerable excess of this precursor. Similar observations on the ideal reagent stoichiometry have been made by Öhberg and Westman [68] in sealed tube microwave reactions and our yields were broadly comparable although higher than our previous report which included an additional purification step [70] (e.g. 70% (Table 2, entry 3) vs 84% [67] or 47% [70]). The use of NH_4_OAc as ammonia source in EtOH–AcOH, under similar conditions to a 3-component Bohlmann–Rahtz reaction [54], failed to improve the efficiency of the process (Table 2, entries 7 and 8) and so, given the high yield and short reaction times of the Westman conditions (Table 2, entries 3 and 4), and the Leadbeater precedent [73], it was felt that this process was suitable for direct transfer to continuous flow processing under conductive heating to examine if this offered any improvement over Leadbeater’s established flow chemistry protocol. Thus, a solution of NH_4_OH, as the ammonia source, aldehyde **14a** and acetoacetate **13** (5 equiv) in EtOH was heated at 140 °C in a 5 mL stainless steel coil for a residence time of 10 min (Table 2, entry 9); the outflow was quenched in H_2_O, extracted and purified using column chromatography [71]. Although the isolated yield of DHP **15a** was lower, in relation to the corresponding batch process, the continuous flow process was a success. Further optimization, by lowering the flow rate and decreasing the reaction time (Table 2, entries 10 and 11), caused a small reduction in the yield of both DHP **15a** and **15b**, which was improved little by increasing the flow rate and thus decreasing the residence time (Table 2, entry 12). However, returning to the original conditions (Table 2, entry 13) delivered a good yield of DHP **15b** (68%) under flow processing. Comparing the optimum conditions under continuous flow processing for this reaction, i.e. NH_4_OH/EtOH/140 °C/10 min, with Leadbeater’s process [73] for this transformation (43% yield vs 53% conversion), it was apparent that the small reduction in efficiency we had observed was reasonably well justified: the change in solvent had prevented problems with in-line precipitation and so greatly simplified the processing protocol. However, the transfer to a continuous flow regime had caused a significant reduction in yield with respect to our microwave batch reaction (Table 2, entry 3; 70% yield) and was considerably lower than the batch microwave process reported by Leadbeater on 0.5 mol scale under open vessel conditions, which delivered an outstanding yield of **15a** (96%) [10], so further experiments to improve the continuous flow processing of Hantzsch dihydropyridines were considered.

**Table 2:** Batch and flow experiments for 4-component Hantzsch DHP **15** synthesis.

Entry	Process	Heating	Reagents and conditions^a^	**15**	Yield^b^

1	Batch	Microwaves^c^	**14a** (1 equiv), **13** (5 equiv), NH_4_OH (4 equiv.), EtOH–H_2_O, 140 °C, 10 min	**15a**	41%
2	Batch	Microwaves^c^	**14b** (1 equiv), **13** (5 equiv), NH_4_OH (4 equiv.), EtOH–H_2_O, 140 °C, 10 min	**15b**	67%
3	Batch	Microwaves^c^	**14a** (1 equiv), **13** (5 equiv), NH_4_OH (4 equiv.), EtOH, 140 °C, 10 min	**15a**	70%
4	Batch	Microwaves^c^	**14b** (1 equiv), **13** (5 equiv), NH_4_OH (4 equiv), EtOH, 140 °C, 10 min	**15b**	82%
5	Batch	Microwaves^c^	**14a** (1 equiv), **13** (3.4 equiv), NH_4_OH (4 equiv), EtOH–H_2_O, 140 °C, 10 min	**15a**	35%
6	Batch	Microwaves^c^	**14b** (1 equiv), **13** (3.4 equiv), NH_4_OH (4 equiv), EtOH–H_2_O, 140 °C, 10 min	**15b**	46%
7	Batch	Microwaves^c^	**14a** (1 equiv), **13** (2 equiv), NH_4_OAc (3 equiv), EtOH–AcOH, 140 °C, 10 min	**15a**	43%
8	Batch	Microwaves^c^	**14b** (1 equiv), **13** (3.4 equiv), NH_4_OAc (6 equiv), EtOH–AcOH, 140 °C, 10 min	**15b**	28%
9	Flow	Conductive^e^	**14a** (1 equiv), **13** (5 equiv), NH_4_OH (4 equiv), EtOH, 140 °C, 10 min [71]	**15a**	43%
10	Flow	Conductive^e^	**14a** (1 equiv), **13** (5 equiv), NH_4_OH (4 equiv), EtOH, 120 °C, 30 min	**15a**	35%
11	Flow	Conductive^e^	**14b** (1 equiv), **13** (5 equiv), NH_4_OH (4 equiv), EtOH, 120 °C, 30 min	**15b**	34%
12	Flow	Conductive^e^	**14b** (1 equiv), **13** (5 equiv), NH_4_OH (4 equiv), EtOH, 140 °C, 7.5 min	**15b**	39%
13	Flow	Conductive^e^	**14b** (1 equiv), **13** (5 equiv), NH_4_OH (4 equiv), EtOH, 140 °C, 10 min	**15b**	68%

^a^Temperature refers to vessel temperature, maintained by moderation of the initial microwave power, as measured by the in-built IR sensor (entries 1–5); ^b^isolated yield of DHP **15** after purification by column chromatography on silica, eluting with EtOAc–light petroleum; ^c^carried out using a commercial CEM single-mode instrument at an initial power of 150 W; ^d^unreacted starting materials were present; ^e^carried out using a commercial Uniqsis FlowSyn stainless steel coil reactor (5 mL) at a flow rate of 0.5 mL min^–1^.

**Scheme 5:**
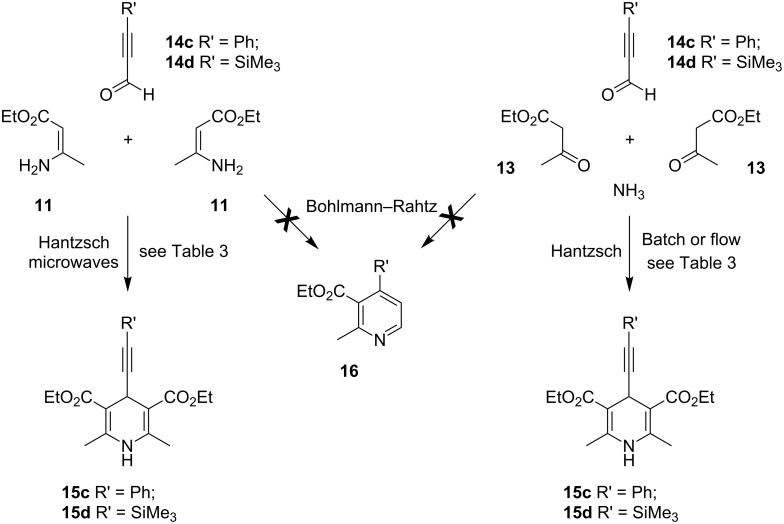
Three- or four-component synthesis of Hantzsch DHP **15c,d**.

In an effort to improve the flow process further, a 3-component process was investigated. In this transformation, the use of an ammonia source was no longer necessary and instead acetoacetate **13** was replaced with ethyl β-aminocrotonate (**11**) (Scheme 5). Removing the need to generate the enamine in situ should improve the efficiency of the process and it was thought could lead to better transfer of reaction parameters between batch and flow platforms. The use of propynal **14c** would further expand the scope of this reaction and establish if 3-substituted propargyl aldehydes would undergo Hantzsch DHP **15c** synthesis or participate instead in a tandem Michael addition–cyclodehydration reaction, in accordance with the original Bohlmann–Rahtz report (which had used propynal), to give trisubstituted pyridines **16** [49]. Returning to the microwave batch reactor, a solution of phenylpropargyl aldehyde (**14c**) and enamine **11** (2 equiv) in PhMe–AcOH (Table 3, entry 1) or EtOH–AcOH (5:1) was irradiated at 100 °C for 1 min, cooled and then extracted and purified as before to give DHP **15c** in a remarkable 98 or >98% yield, respectively. Clearly, using stoichiometry appropriate for Hantzsch DHP synthesis, this process was totally selective over Bohlmann–Rahtz pyridine synthesis and no 2,3,4-trisubstituted pyridine **16** was formed (Scheme 5). This supported earlier findings [49] by Bohlmann and Rahtz and highlights a reactivity trend of 3-substituted propargyl aldehydes in reaction with enamines. A comparable process in EtOH in the absence of AcOH failed to provide complete conversion (Table 3, entry 3), whereas the reagents were only poorly soluble in AcOH alone and so the effect of change in solvent was not pursued further. To try and identify which component, aldehyde or acetoacetate, had been responsible for the dramatic improvement in yield, a 4-component Hantzsch reaction was also investigated (Table 3, entry 4). Irradiating a solution of propargyl aldehyde **14c**, acetoacetate **13** (2 equiv) and NH_4_OAc (3 equiv) in EtOH–AcOH (5:1) at 120 °C for 5 min gave DHP **15c** in 96% isolated yield. Investigating an alternative propargyl aldehyde, the 3- or 4-component batch syntheses of DHP **15d** using 3-(trimethylsilyl)propynal (**14d**), similarly, gave excellent yields of the product under microwave irradiation (Table 3, entries 5 and 6). Thus, it was concluded that 3-substituted propargyl aldehydes are highly reactive and useful substrates for Hantzsch DHP synthesis and give little or no competing formation of the corresponding Bohlmann–Rahtz pyridine **16** under conditions that nominally can promote both processes.

**Table 3:** Synthesis of Hantzsch DHP **15c,d** from propargyl aldehydes **14c,d** in batch and flow.

Entry	Process	Heating	Reagents and conditions^a^	**15**	Yield^b^

1	Batch	Microwaves^c^	**14c** (1 equiv), **11** (2 equiv), PhMe–AcOH, 100 °C, 1 min	**15c**	98%
2	Batch	Microwaves^c^	**14c** (1 equiv), **11** (2 equiv), EtOH–AcOH, 100 °C, 1 min	**15c**	>98%
3	Batch	Microwaves^c^	**14c** (1 equiv), **11** (2 equiv), EtOH, 120 °C, 15 min	**15c**	–^d^
4	Batch	Microwaves^c^	**14c** (1 equiv), **13** (2 equiv), NH_4_OAc (3 equiv), EtOH–AcOH, 120 °C, 5 min	**15c**	96%
5	Batch	Microwaves^c^	**14d** (1 equiv), **11** (2 equiv), EtOH–AcOH, 100 °C, 1 min	**15d**	82%
6	Batch	Microwaves^c^	**14d** (1 equiv), **13** (2 equiv), NH_4_OAc (3 equiv), EtOH–AcOH, 120 °C, 5 min	**15d**	84%
7	Flow^e^	Microwaves^c^	**14d** (1 equiv), **13** (2 equiv), NH_4_OAc (3 equiv), PhMe–AcOH, 120 °C, 5 min	**15d**	–^f^
8	Flow^e^	Microwaves^c^	**14c** (1 equiv), **13** (2 equiv), NH_4_OAc (3 equiv), EtOH–AcOH, 120 °C, 5 min	**15c**	70%
9	Flow^e^	Microwaves^c^	**14c** (1 equiv), **13** (2 equiv), NH_4_OAc (3 equiv), EtOH–AcOH, 120 °C, 5 min	**15c**	85%^g^

^a^Temperature refers to vessel temperature, maintained by moderation of the initial microwave power, as measured by the in-built IR sensor; ^b^isolated yield of DHP **15** after quenching in H_2_O and extraction (entries 1–8); ^c^carried out using a commercial CEM single-mode instrument at an initial power of 70 W (entries 1–3 and 5), 90 W (entries 4 and 6), 200 W (entry 7) or 100 W (entries 8 and 9); ^d^unreacted starting materials were present; ^e^carried out using the microwave flow reactor (10 mL) filled with sand at a flow rate of 0.6 mL min^−1^; ^f^heterogeneity in the solvent system caused pump failure; ^g^isolated yield after quenching in aqueous NaHCO_3_ solution and filtering the precipitated solid.

With the batch methods established, a 4-component reaction using a propargyl aldehyde was transferred to the microwave flow reactor with minimal change in reaction parameters. The flow cell was primed with the solvent of choice and heated under microwave irradiation; once the temperature of the reactor stabilized, the reaction mixture was introduced. Using 3-(trimethylsilyl)propynal (**14d**) in PhMe–AcOH resulted in pump failure, due to the heterogeneity of the reagent flow in this reaction solvent (Table 3, entry 7). Switching to the use of phenylpropargyl aldehyde (**14c**) and changing the solvent system to EtOH–AcOH produced a homogeneous reagent flow and allowed the reaction mixture to be processed at 120 °C at a continuous flow rate of 0.5 mL min^−1^ through the microwave reactor. After passing through the back-pressure regulator, the outflow was quenched in H_2_O and extracted (Table 3, entry 8) or quenched in aqueous NaHCO_3_ solution and filtered (Table 3, entry 9) to give the 4-(phenylethynyl)-DHP **15c** in 70 or 85% yield, respectively. Although the yields of both flow reactions were slightly lower than their batch mode counterparts, (96% batch yield vs 85% yield under flow processing for the synthesis of **15c**) the continuous processing of Hantzsch DHPs had been realized.

Reviewing all of our methods for the microwave-assisted preparation of DHP derivatives, the isolated yield for the batch synthesis of Hantzsch DHP **15c** (96%) compares very favourably to other microwave-assisted 4-component Hantzsch reactions (cf. 81% yield of a DHP under flow processing [74], 51–92% yield [67] or 84–99% yield [72] of a range of derivatives in batch using a single-mode instrument, and 96% yield of **15a** under open vessel batch conditions on 0.5 mol scale [10]) and transfers well to flow processing giving 85% yield under microwave irradiation (cf. 81% [74]). Given the excellent performance of microwave dielectric heating in promoting the 4-component Hantzsch reaction with direct scalability under microwave irradiation [10] and under continuous flow processing, observed by ourselves and others [73–74], this technology stands out as the heating method of choice for the preparation of 1,4-DHP derivatives.

## Conclusion

These studies have demonstrated that a microwave flow reactor can be used for the one-step preparation of pyridines and dihydropyridines using the Bohlmann–Rahtz reaction or Hantzsch multicomponent reaction, respectively. Bohlmann–Rahtz pyridine synthesis under continuous flow processing in the presence of a Brønsted acid catalyst allows Michael addition and cyclodehydration to be carried out in one step without the isolation of intermediates to give a trisubstituted pyridine as a single regioisomer. Furthermore, the use of microwave heating for facilitating this two-step-in-one transformation is well justified, compares favourably with the traditional two-step procedure and, using these protocols, delivers Bohlmann–Rahtz pyridines quickly and efficiently. In batch mode using a single-mode instrument this process is highly predictable and is most reliably scaled up using continuous flow processing, either on a conductive heating platform or using a microwave flow reactor in favour over a multimode batch reactor. On the other hand, the scale up of a microwave-assisted Hantzsch DHP synthesis under open-vessel conditions as described by Leadbeater [10] outperforms even the small-scale microwave-assisted batch reaction, but the use of continuous flow processing in a microwave reactor as shown by ourselves and others [74] can deliver the target heterocycle in excellent yield. For both Hantzsch and Bohlmann–Rahtz reactions, parameters transferred very well from high temperature batch conditions in a sealed vessel to continuous flow processing through a microwave flow cell in a single-mode cavity. In some cases, it was possible to further transfer parameters between conductive heating and microwave heated flow platforms, with only minor variations in yield. Furthermore, it has been affirmed that 3-substituted propargyl aldehydes are not suitable substrates for the Bohlmann–Rahtz reaction and instead undergo Hantzsch dihydropyridine synthesis in very high yield in a process that is readily transferred to continuous flow processing in a microwave flow cell. Although this sets a new challenge on how to access 2,3,4-trisubstituted pyridines using Bohlmann–Rahtz methods, a transformation which currently cannot be realized, it does provide a useful substrate for 3- or 4-component Hantzsch DHP synthesis that undergoes cyclocondensation with high efficiency. To conclude, continuous flow microwave-assisted reactions represent a reliable method to scale up the production of pyridine derivatives and, for the Bohlmann–Rahtz pyridine synthesis, give improved performance over a comparable large scale multimode batch experiment. This expands the growing set of heterocyclic targets that have been accessed by the reactions of ethynyl ketones under continuous flow processing and sets the stage for their future incorporation into automated multistep processes.

## Experimental

### Diethyl 4-(trimethylsilylethynyl)-2,6-dimethyl-1,4-dihydropyridine-3,5-dicarboxylate (**15d**)


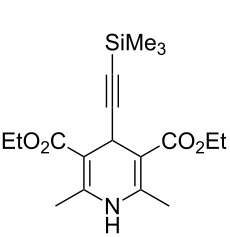


**3-Component Hantzsch DHP synthesis in a single-mode microwave batch reactor** (Table 3, entry 5). A mixture of 3-(trimethylsilyl)propynal (**14d**, 50 mg, 0.53 mmol) and ethyl β-aminocrotonate (**11**, 0.14 g, 1.1 mmol) in PhMe–glacial acetic acid (5:1, 2 mL) was irradiated at 100 °C for 1 min in a sealed tube using a CEM Discover microwave synthesizer at an initial power of 70 W. The reaction mixture was cooled in a stream of compressed air and partitioned between saturated aqueous NaHCO_3_ solution (25 mL) and EtOAc (25 mL). The aqueous layer was further extracted with EtOAc (2 × 15 mL) and the combined organic extracts were washed with brine (15 mL), dried (NaSO_4_) and evaporated in vacuo to give the title compound (0.15 g, 82%) as a yellow solid, mp 137–138 °C (aq EtOH); (Found: [M + H]^+^, 350.1783. C_18_H_27_NO_4_Si, [M + H] requires 350.1782); *R*_f_ 0.47 (light petroleum–EtOAc, 1:1); IR (nujol)/cm^−1^: 3302, 3244, 3107, 1699, 1661, 1636, 1503, 1328, 1301, 1208, 1120, 1095, 1026, 840; ^1^H NMR (400 MHz, CDCl_3_) δ 5.69 (br s, 1H, NH), 4.72 (s, 1H, 4-H), 4.11 (m, 2H, OC*H*HCH_3_), 4.08 (m, 2H, OCH*H*CH_3_), 2.20 (s, 6H, 2,6-CH_3_), 1.21 (t, *J* 7.1, 6H, OCH_2_C*H*_3_), 0.00 (s, 9H, SiMe_3_); ^13^C NMR (100 MHz, CDCl_3_) δ 167.0 (C), 144.9 (C), 109.8 (C), 100.2 (C), 82.5 (C), 59.8 (CH_2_), 27.6 (CH), 19.5 (CH_3_), 14.4 (CH_3_), 0.22 (CH_3_); MS (APcI) *m/z* (rel intensity): 350 (MH^+^, 100%), 252 (15), 178 (15), 113 (10).

**4-Component Hantzsch DHP synthesis in a single-mode microwave batch reactor** (Table 3, entry 6). A solution of 3-(trimethylsilyl)propynal (**14d**) (50 mg, 0.53 mmol), ethyl acetoacetate (**11**) (0.14 g, 1.1 mmol) and ammonium acetate (0.12 g, 1.6 mmol) in EtOH–glacial acetic acid (5:1, 2 mL) was irradiated at 120 °C for 7 min in a sealed tube using a CEM Discover microwave synthesizer at an initial power of 90 W. The reaction mixture was cooled in a stream of compressed air and evaporated in vacuo. The residue was partitioned between saturated aqueous NaHCO_3_ solution (25 mL) and CH_2_Cl_2_ (25 mL). The aqueous layer was further extracted with CH_2_Cl_2_ (2 × 15 mL) and the organic extracts were combined, washed with brine (15 mL), dried (NaSO_4_) and evaporated in vacuo to give the title compound (0.16 g, 84%) as a pale yellow solid, with identical physical and spectroscopic properties.

## Supporting Information

Supporting information contains experimental procedures for the synthesis of known compounds.

File 1 General experimental methods and detailed procedures for the synthesis of propynone **12b**, Bohlmann–Rahtz pyridine **2b** and Hantzsch dihydropyridines **15a**, **15b** and **15c**.
