# Supplementary material for: One-step synthesis of pyridines and dihydropyridines in a continuous flow microwave reactor
Source: Beilstein J Org Chem. 2013 Sep 30;9:1957–68. doi: 10.3762/bjoc.9.232 (PMC3817555; doi:10.3762/bjoc.9.232)
Supplement: File 1 — General experimental methods and detailed procedures for the synthesis of propynone 12b, Bohlmann–Rahtz pyridine 2b and Hantzsch dihydropyridines 15a, 15b and 15c. [file Beilstein_J_Org_Chem-09-1957-s001.pdf]

Supporting Information

for

One-step synthesis of pyridines and dihydropyridines in a continuous flow microwave reactor

Mark C. Bagley*[1], Vincenzo Fusillo[2], Robert L. Jenkins[2], M. Caterina Lubinu[2] and Christopher Mason[3]

Address: [1]Department of Chemistry, School of Life Sciences, University of Sussex, Falmer, Brighton, East Sussex, BN1 9QJ, UK, [2]School of Chemistry, Main Building, Cardiff University, Park Place, Cardiff, CF10 3AT, UK and [3]CEM Microwave Technology Ltd, 2 Middle Slade, Buckingham, MK18 1WA, UK

Email: Mark Christopher Bagley - m.c.bagley@sussex.ac.uk

* Corresponding author

General experimental methods and detailed procedures for the synthesis of propynone 12b, Bohlmann–Rahtz pyridine 2b and Hantzsch dihydropyridines 15a, 15b and 15c.

S1 General experimental methods
S3 Procedures for Bohlmann-Rahtz synthesis of pyridines
1-Phenylprop-2-yn-1-one (**12b**)
Ethyl 2-methyl-6-phenylpyridine-3-carboxylate (**2b**)
S6 Procedures for Hantzsch dihydropyridine synthesis
Diethyl 4-phenyl-2,6-dimethyl-1,4-dihydropyridine-3,5-dicarboxylate (**15a**)
S7 Diethyl 4-ethyl-2,6-dimethyl-1,4-dihydropyridine-3,5-dicarboxylate (**15b**)
S8 Diethyl 4-(phenylethynyl)-2,6-dimethyl-1,4-dihydropyridine-3,5-dicarboxylate (**15c**)
S11 References

Experimental

General experimental methods

Commercially available reagents were used without further purification and solvents were dried by standard procedures. Light petroleum refers to the fraction with bp 40–60 °C and ether refers to diethyl ether. Microwave irradiation experiments were performed at the given temperature by varying the irradiation power (initial power given in parentheses) under an atmosphere of air using a self-tunable single-mode CEM Discover microwave synthesizer, operating in batch or continuous flow mode according to our reported method [1] with temperature measurement by the in-built IR sensor, or using a MILESTONE multi-mode BatchSynth^{TM} microwave synthesizer, with temperature measurement by thermocouple. Conductive heating flow reactions were performed using the Uniqsis FlowSyn™ using the instrument's in-built temperature measurement. Analytical thin layer chromatography was carried out using aluminium-backed plates coated with Merck Kieselgel 60 GF_{254} that were visualised under UV light (at 254 and/or 360 nm). In vacuo refers to evaporation at reduced pressure using a rotary evaporator and diaphragm pump, followed by the removal of trace volatiles using a vacuum (oil) pump. Fully characterized compounds were chromatographically homogeneous. Melting points were determined on a Kofler hot stage apparatus and are uncorrected. Infra-red spectra were recorded in the range 4000–600 cm^{-1} on a Perkin-Elmer 1600 series FTIR spectrometer using KBr disks or as a nujol mull for solid samples and thin films between NaCl plates for liquid samples and are reported in cm^{-1}. NMR spectra were recorded using a Bruker DPX 400 instrument or 500 Avance instrument operating at 400 MHz for ^{1}H spectra and 100 or 125 MHz for ^{13}C spectra and were reported in ppm; *J* values were recorded in Hz and multiplicities were expressed by the usual conventions. Low resolution mass spectra were determined using a Fisons

VG Platform II Quadrupole instrument using atmospheric pressure chemical ionization (APcI) or electrospray (ES) unless otherwise stated. High resolution mass spectra were obtained courtesy of the EPSRC Mass Spectrometry Service at University College of Wales, Swansea, UK using the ionization methods specified.

Procedures for Bohlmann–Rahtz synthesis of pyridines

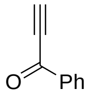

12b

1-Phenylprop-2-yn-1-one (12b). In a similar way as described previously [2], a solution of *o*-iodoxybenzoic acid (IBX) (6.6 g, 24 mmol) in DMSO (350 mL) was stirred for 15 min at room temperature until homogeneous. A solution of 1-phenylprop-2-yn-1-ol (2.4 g, 18 mmol) in DMSO (10 mL) was added and the mixture was stirred for 16 h. H_2O (35 mL) was added and the mixture was stirred at room temperature for 10 min, cooled in ice and partitioned between H_2O (290 mL) and Et_2O (210 mL). The mixture was filtered through Celite and the aqueous layer was further extracted with Et_2O (170 mL). The organic extracts were combined, washed sequentially with H_2O (3 x 170 mL), saturated aqueous $NaHCO_3$ (230 mL) and brine (230 mL), dried ($MgSO_4$) and evaporated in vacuo to give the title compound (2.0 g, 84%) as a pale yellow solid, mp 48–50 °C (MeOH) (lit.[2] mp 49–50 °C) (Found: $M^{\bullet+}$, 130.0411. C_9H_6O [*M*] requires 130.0413); R_f 0.44 (CH_2Cl_2); IR (nujol)/cm^{-1} 1647, 1591, 1583, 1452, 1314, 1269, 1170, 1004, 696; ^{1}H NMR (400 MHz; $CDCl_3$) δ 8.09 (2H, m, *o*-Ph*H*), 7.56 (1H, m, *p*-Ph*H*), 7.40 (2H, m, *m*-Ph*H*), 3.37 (1H, s, CH); ^{13}C NMR (100 MHz; $CDCl_3$) δ 177.5 (C), 136.1 (C), 134.6 (CH), 129.7 (CH), 128.7 (CH), 80.9 (C), 80.3 (CH); *m/z* (EI) 130 ($M^{\bullet+}$, 156%), 102 (21), 77 (32), 53 (100), 51 (40).

Ethyl 2-methyl-6-phenylpyridine-3-carboxylate (2b)

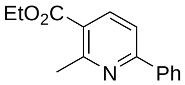

2b

Bohlmann–Rahtz pyridine synthesis in a single-mode microwave batch reactor (Table 1, entry 4). In a way as described previously [3], a solution of ethyl β-aminocrotonate (**11**) (52 mg, 0.40 mmol) and 1-phenylprop-2-yn-1-one (**12b**) (40 mg, 0.31 mmol) in EtOH–glacial acetic acid (5:1; 3 mL) was irradiated at 120 °C for 5 min in a sealed Pyrex tube (10 mL) using a CEM Discover microwave synthesizer at an initial power of 90 W. The solution was cooled in a flow of compressed air and evaporated in vacuo. The residue was partitioned between saturated aqueous $NaHCO_3$ solution (25 mL) and EtOAc (25 mL) and the aqueous layer was further extracted with EtOAc (2 x 15 mL). The organic extracts were combined, washed with brine (15 mL), dried (Na_2SO_4) and evaporated in vacuo. Purification by column chromatography on SiO_2, eluting with light petroleum–EtOAc (8:2), gave the title compound (64 mg, 86%) as a pale yellow solid, mp 44–45 °C (aq. EtOH) (lit.[4] mp 44 °C) (Found: MH^+, 242.1176. $C_{15}H_{16}NO_2$ [*MH*] requires 242.1176); R_f 0.47 (light petroleum–EtOAc, 8:2); IR (nujol)/cm^{-1} 1717, 1581, 1476, 1277, 1090 and 1022; ^1H NMR (400 MHz; $CDCl_3$) δ 8.21 (1H, d, *J* 8.2, 4-H), 8.00 (2H, m, *o*-PhH), 7.59 (1H, d, *J* 8.2, 5-H), 7.41 (3H, *m.p*-PhH), 4.28 (2H, q, *J* 7.1, OCH_2CH_3), 2.85 (3H, s, CH_3), 1.35 (3H, t, *J* 7.1, OCH_2CH_3); ^{13}C NMR (100 MHz; $CDCl_3$) δ 165.7 (C), 159.1 (C), 158.1 (C), 138.5 (CH), 137.6 (C), 128.7 (CH), 128.0 (CH), 127.6 (CH),122.8 (C), 116.5 (CH), 60.3 (CH_2), 24.6 (CH_3), 13.5 (CH_3); MS (APcI) *m/z* (rel. intensity) 242 (MH^+, 100%), 214 (50).

Bohlmann–Rahtz pyridine synthesis in a microwave flow reactor (Table 1, entry 5). A pressure-rated glass tube flow cell (10 mL) was filled with sand (~12 g), primed with EtOH–glacial acetic acid (5:1) at a flow rate of 0.6 mL/min using a back-pressure regulator (100 psi), and irradiated at 120 °C at an initial power of 100 W. Once the

temperature stabilized at 120 °C, a flask was charged with a solution of ethyl β-aminocrotonate (**11**) (52 mg, 0.40 mmol) and 1-phenylprop-2-yn-1-one (**12b**) (40 mg, 0.31 mmol) in EtOH–glacial acetic acid (5:1; 3 mL). In a way as described previously [3], the solution was then passed through the cavity at the given flow rate, washing with further batches of solvent. The reaction mixture was quenched in a solution of saturated aqueous $NaHCO_3$, extracted with dichloromethane (3 x 30 mL), dried ($NaSO_4$) and evaporated in vacuo. Purification by column chromatography on SiO_2, eluting with light petroleum–EtOAc (8:2), gave the title compound (56 mg, 76%) as a pale yellow solid, with identical physical and spectroscopic properties.

Bohlmann–Rahtz pyridine synthesis in a conductive heating flow reactor (Table 1, entry 6). In a way as described previously [3], a solution of 1-phenylprop-2-yn-1-one (**12b**) (0.16 g, 1.2 mmol) and ethyl β-aminocrotonate (**11**) (0.20 mL, 1.6 mmol) in EtOH–glacial acetic acid (5:1; 12 mL) was passed through a steel tubing reactor cassette (5 mL) at 120 °C and a flow rate of 1 mL min^{-1} using a UNIQSIS FlowSyn™ reactor. The outflow from the collection valve was directed into a collection bottle containing a stirred solution of saturated aqueous $NaHCO_3$ and extracted with EtOAc (3 x 10 mL). The organic extracts were combined, dried ($MgSO_4$) and evaporated in vacuo to give the title compound (0.25 g, 86%) as a yellow solid, mp 44–45 °C (lit.[3] mp 44 °C), with identical physical and spectroscopic properties.

Bohlmann–Rahtz pyridine synthesis scaled-up in a multi-mode microwave batch reactor (Table 1, entry 8). A solution of 1-phenylprop-2-yn-1-one (**12b**) (0.50 g, 15 mmol) and ethyl β-aminocrotonate (**11**) (2.6 mL, 21 mmol) in EtOH–glacial acetic acid (5:1; 38 mL) was irradiated at 100 °C (150 W) for 5 min in a pressure-rated Teflon vessel (60 mL) using a multi-mode MILESTONE BatchSynth™ microwave synthesizer. The mixture was quenched immediately in a solution of saturated aqueous $NaHCO_3$ and extracted with EtOAc (3 x 30 mL). The organic extracts were combined, dried

($MgSO_4$) and evaporated in vacuo. Purification by column chromatography on SiO_2, eluting with light petroleum–EtOAc (4:1), gave the title compound (1.2 g, 32%) as a yellow solid, mp 43–44 °C (MeOH) (lit.[3] mp 44 °C), with identical physical and spectroscopic properties.

Procedures for Hantzsch dihydropyridine synthesis

Diethyl 4-phenyl-2,6-dimethyl-1,4-dihydropyridine-3,5-dicarboxylate (15a)

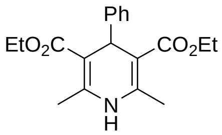

15a

4-Component Hantzsch DHP synthesis in a single-mode microwave batch reactor (Table 2, entry 3). In a similar way as described previously [5-7], a mixture of benzaldehyde (**14a**) (0.26 g, 2.5 mmol), ethyl acetoacetate (**13**) (1.6 mL, 12.5 mmol) and 35% aqueous ammonium hydroxide (0.55 mL, 10 mmol) in EtOH (1.4 mL) was irradiated at 140 °C for 10 min in a sealed tube using a CEM Discover microwave synthesizer by moderating the initial power (150 W). After cooling in a stream of compressed air, the mixture was evaporated in vacuo. Purification by column chromatography on SiO_2, eluting with light petroleum–EtOAc (7:1), gave the title compound (0.58 g, 70%) as a pale yellow solid, mp 158–160 °C (aq. EtOH) (lit.[8] mp 156–157 °C) (Found: MH^+, 330.1700. $C_{19}H_{24}NO_4$, [*MH*] requires 330.1700); IR (nujol)/cm^{-1} 3340, 1688, 1650, 1298, 1212, 1124, 1090, 1018, 827; ^{1}H NMR (400 MHz; $CDCl_3$) δ 7.23–7.03 (5H, PhH), 5.48 (1H, br s, NH), 4.92 (1H, s, 4-H), 4.02 (4H, m, CH_2), 2.27 (6H, s, 2,6-Me), 1.15 (6H, t, *J* 7.1, CH_2*Me*); ^{13}C NMR (100 MHz; $CDCl_3$) δ 167.6 (C), 147.7 (C), 143.7 (C), 128.0 (CH), 127.8 (CH), 126.1 (CH), 104.3 (C), 59.7 (CH_2), 39.6 (CH), 19.6 (CH_3), 14.2 (CH_3); MS (APcI) *m/z* (rel. intensity) 330 (MH^+, 100%), 284 (44).

4-Component Hantzsch DHP synthesis in a conductive heating flow reactor (Table 2, entry 9). In a way as described previously [6], a mixture of benzaldehyde (**14a**) (0.26 g, 2.5 mmol), ethyl acetoacetate (**13**) (1.6 mL, 12.5 mmol) and 35% aqueous ammonium hydroxide (0.55 mL, 10 mmol) in EtOH (1 mL) was passed through a steel tubing reactor cassette (5 mL) at 140 °C and a flow rate of 0.5 mL min^{-1} using a UNIQSIS FlowSyn™ reactor. The outflow from the collection valve was poured into cold water (10 mL) and extracted with EtOAc. The organic extracts were combined, dried ($MgSO_4$) and evaporated in vacuo. Purification by column chromatography on SiO_2, eluting with light petroleum–EtOAc (7:1), gave the title compound (0.34 g, 43%) as a pale yellow solid, with identical physical and spectroscopic properties.

Diethyl 4-ethyl-2,6-dimethyl-1,4-dihydropyridine-3,5-dicarboxylate (15b)

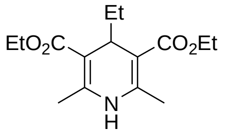

15b

4-Component Hantzsch DHP synthesis in a single-mode microwave batch reactor (Table 2, entry 4). In a way as described previously [6], a mixture of propionaldehyde (**14b**) (0.18 mL, 2.5 mmol), ethyl acetoacetate (**13**) (1.6 mL, 12.5 mmol) and 35% aqueous ammonium hydroxide (0.55 mL, 10 mmol) in EtOH (1.4 mL) was irradiated for 10 min at 140 °C in a sealed tube using a CEM Discover microwave synthesizer by moderating the initial power (150 W). After cooling in a stream of compressed air, the mixture was evaporated in vacuo. Purification by column chromatography on SiO_2, eluting with light petroleum–EtOAc (8:2), gave the title compound (0.58 g, 82%) as pale yellow solid, mp 111–112 °C (aq. EtOH) (lit.[9] mp 110 °C) (Found: MH^+, 282.1699. $C_{15}H_{24}NO_4$ [*MH*] requires 282.1700); IR (nujol)/cm^{-1} 3312, 1699, 1651, 1302, 1211, 1133, 1072, 999; ^{1}H NMR (400 MHz; $CDCl_3$) δ 5.38 (1H, br s, NH), 4.17–4.04 (4H, m,

OCH_2), 3.84 (1H, t, *J* 5.5, 4-H), 2.22 (6H, s, 2,6-Me), 1.29 (2H, m, CH_2), 1.23 (6H, t, *J* 7.1, OCH_2*Me*), 0.69 (3H, t, *J* 7.5, CH_2*Me*); ^{13}C NMR (100 MHz; $CDCl_3$) δ 168.1 (C), 144.6 (C), 102.8 (C), 59.5 (CH_2), 34.1 (CH), 29.3 (CH_2), 19.5 (CH_3), 14.4 (CH_3), 9.2 (CH_3); MS (ES) *m/z* (rel. intensity) 282 (MH^+, 18%), 236 (100).

4-Component Hantzsch DHP synthesis in a conductive heating flow reactor (Table 2, entry 13). A mixture of propionaldehyde (**14b**) (0.18 mL, 2.5 mmol), ethyl acetoacetate (**13**) (1.6 mL, 12.5 mmol) and 35% aqueous ammonium hydroxide (0.55 mL, 10 mmol) in EtOH (1 mL) was passed through a steel tubing reactor cassette (5 mL) at 140 °C and a flow rate of 0.5 mL min^{-1} using a UNIQSIS FlowSyn™ reactor. The outflow from the collection valve was poured into cold water (10 mL) and extracted with EtOAc. The organic extracts were combined, dried ($MgSO_4$) and evaporated in vacuo. Purification by column chromatography on SiO_2, eluting with light petroleum–EtOAc (4:1), gave the title compound (0.48 g, 68%) as a pale yellow solid, with identical physical and spectroscopic properties.

Diethyl 4-(phenylethynyl)-2,6-dimethyl-1,4-dihydropyridine-3,5-dicarboxylate (15c)

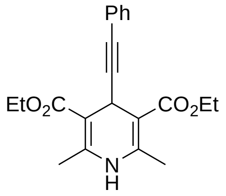

15c

3-Component Hantzsch DHP synthesis in a single-mode microwave batch reactor (Table 3, entries 1 and 2). A mixture of phenylpropargyl aldehyde (**14c**) (65 µL, 0.53 mmol) and ethyl β-aminocrotonate (**11**) (0.14 g, 1.1 mmol) in PhMe–glacial acetic acid (5:1; 2 mL) was irradiated at 100 °C for 1 min in a sealed tube using a CEM Discover microwave synthesizer at an initial power of 70 W. The reaction mixture was cooled in stream of compressed air and partitioned between saturated aqueous $NaHCO_3$ solution (25 mL) and EtOAc (25 mL). The aqueous layer was further extracted with ethyl acetate

(2 x 15 mL) and the combined organic extracts were washed with brine (15 mL), dried ($NaSO_4$) and evaporated in vacuo to give the title compound (0.18 g, 98%) as a yellow solid, mp 190–192 °C (aq. EtOH) (lit.[4] mp 192 °C) (Found: MH^+, 354.1700. $C_{21}H_{24}NO_4$, [*MH*] requires 354.1696); R_f 0.39 (light petroleum–EtOAc, 1:1); IR (nujol)/cm^{-1} 3336, 1693, 1649, 1329, 1299, 1215, 1124, 1095, 761, 693; ^{1}H NMR (400 MHz; $CDCl_3$) δ 7.35 (2H, m, *o*-PhH), 7.18 (3H, m, *m,p*-PhH), 5.67 (1H, br s, NH), 5.20 (1H, s, 4-H), 4.32–4.10 (4H, m, OCH_2CH_3), 2.28 (6H, s, CH_3), 1.27 (6H, t, *J* 7.1, OCH_2CH_3); ^{13}C NMR (100 MHz; $CDCl_3$) δ 167.1 (C), 145.1 (C), 131.7 (CH), 128.0 (CH), 127.4 (CH), 124.1 (C), 100.3 (C), 93.1 (C), 79.2 (C), 59.1 (CH_2), 23.1 (CH), 19.6 (CH_3), 14.5 (CH_3), MS (APcI) *m/z* (rel intensity) 354 (MH^+, 52%), 252 (100).

Alternatively, a solution of phenylpropargyl aldehyde (**14c**) (65 µL, 0.53 mmol) and ethyl β-aminocrotonate (**11**) (0.14 g, 1.1 mmol) in EtOH–glacial acetic acid (5:1; 2 mL) was irradiated at 100 °C for 1 min in a sealed tube using a CEM Discover microwave synthesizer at an initial power of 70 W. The reaction mixture was cooled in stream of compressed air and evaporated in vacuo. The residue was partitioned between saturated aqueous $NaHCO_3$ solution (25 mL) and CH_2Cl_2 (25 mL) and the aqueous layer was further extracted with CH_2Cl_2 (2 x 15 ml). The combined organic extracts were washed with brine (15 mL), dried ($NaSO_4$) and evaporated in vacuo to give the title compound (0.19 g, >98%) as a yellow solid, with identical physical and spectroscopic properties.

4-Component Hantzsch DHP synthesis in a single-mode microwave batch reactor (Table 3, entry 4). A solution of phenylpropargyl aldehyde (**14c**) (65 µL, 0.53 mmol), ethyl acetoacetate (**11**) (0.14 g, 1.1 mmol) and ammonium acetate (0.12 g, 1.6 mmol) in EtOH–glacial acetic acid (5:1; 2 mL) was irradiated at 120 °C for 5 min in a sealed tube using a CEM Discover microwave synthesizer at an initial power of 90 W. The reaction mixture was cooled in a stream of compressed air and evaporated in vacuo. The

residue was partitioned between saturated aqueous $NaHCO_3$ solution (25 mL) and CH_2Cl_2 (25 mL) and the aqueous layer was further extracted with CH_2Cl_2 (2 x 15 ml). The combined organic extracts were washed with brine (15 mL), dried ($NaSO_4$) and evaporated in vacuo to give the title compound (0.18 g, 96%) as a yellow solid, with identical physical and spectroscopic properties.

4-Component Hantzsch DHP synthesis in a microwave flow reactor (Table 3, entries 8 and 9). A pressure-rated glass tube flow cell (10 mL) was filled with sand (~12 g), primed with EtOH–glacial acetic acid (5:1) at a flow rate of 0.6 mL min^{-1} using a back-pressure regulator (100 psi), and irradiated at 120 °C at an initial power of 100 W. A flask was charged with a solution of phenylpropargyl aldehyde (**14c**) (65 µL, 0.53 mmol), ethyl acetoacetate (**11**) (0.14 g, 1.1 mmol) and ammonium acetate (0.12 g, 1.6 mmol) in EtOH–glacial acetic acid (5:1; 2 mL), which was then passed through the cavity at the given flow rate, washing with further batches of solvent. The reaction mixture was quenched in a solution of saturated aqueous $NaHCO_3$ and extracted with CH_2Cl_2 (25 mL). The aqueous layer was further extracted with CH_2Cl_2 (2 x 15 ml) and the organic extracts were combined, washed with brine (15 mL), dried ($NaSO_4$) and evaporated in vacuo to give the title compound (0.13 g, 70%) as a yellow solid, with identical physical and spectroscopic properties.

Alternatively, the reaction mixture was quenched in a solution of saturated aqueous $NaHCO_3$ and the precipitated solid was filtered, washed and dried in vacuo to give the *title compound* (0.16 g, 85%) as a yellow solid, with identical physical and spectroscopic properties.

References

1. Bagley, M. C.; Jenkins, R. L.; Lubinu, M. C.; Mason, C.; Wood, R. *J. Org. Chem.*, **2005**, *70*, 7003–7006.

2. Bagley, M. C.; Brace, C.; Dale, J. W.; Ohnesorge, M.; Phillips, N. G.; Xiong, X.; Bower, J. *J. Chem. Soc., Perkin Trans. 1*, **2002**, 1663-1671.

3. Flow chemistry application note 15: Bohlmann-Rahtz pyridine synthesis, Uniqsis Ltd. http://www.uniqsis.com/fcApplications.aspx (accessed Aug 22, 2013).

4. Bohlmann, F.; Rahtz, D. *Chem. Ber.*, **1957**, *90*, 2265–2272.

5. Öhberg, L.; Westman, J. *Synlett*, **2001**, 1296–1298.

6. Flow chemistry application note 10: Hantzsch dihydropyridine synthesis, Uniqsis Ltd. http://www.uniqsis.com/fcApplications.aspx (accessed Aug 22, 2013).

7. Bagley, M. C.; Lubinu, M. C. *Synthesis*, **2006**, 1283–1288.

8. Schiff, R.; Puliti, J. *Ber. Dtsch. Chem. Ges.*, **1883**, *16*, 1607–1608.

9. Engelman, F. *Justus Liebigs Ann.Chem.*, **1885**, *231*, 37–71.
